# Effects of a popular exercise and weight loss program on weight loss, body composition, energy expenditure and health in obese women

**DOI:** 10.1186/1743-7075-6-23

**Published:** 2009-05-14

**Authors:** Chad Kerksick, Ashli Thomas, Bill Campbell, Lem Taylor, Colin Wilborn, Brandon Marcello, Mike Roberts, Emily Pfau, Megan Grimstvedt, Jasmine Opusunju, Teresa Magrans-Courtney, Christopher Rasmussen, Ron Wilson, Richard B Kreider

**Affiliations:** 1 Health and Exercise Science Department, University of Oklahoma, Norman, Oklahoma 73019-6081, USA; 2 Endocrinology and Diabetes Section, Department of Pediatrics, University of Oklahoma Health Sciences Center, Oklahoma City, Oklahoma 73104, USA; 3 Department of Health, Human Performance and Recreation, Baylor University, One Bear Place, Box 97313, Waco, Texas 76798-7313, USA; 4 School of Physical Education & Exercise Science, University of South Florida, Tampa, Florida 33620, USA; 5 Exercise & Sport Science Department, University of Mary-Hardin Baylor, Belton, Texas 76513, USA; 6 Department of Athletics, Stanford University, Palo Alto, California 94305, USA; 7 Department of Health & Kinesiology, Texas A & M University, College Station, Texas 77843, USA

## Abstract

**Objective:**

To determine the safety and efficacy of altering the ratio of carbohydrate and protein in low-energy diets in conjunction with a popular exercise program in obese women.

**Design:**

Matched, prospective clinical intervention study to assess efficacy of varying ratios of carbohydrate and protein intake in conjunction with a regular exercise program.

**Participants:**

One-hundred sixty one sedentary, obese, pre-menopausal women (38.5 ± 8.5 yrs, 164.2 ± 6.7 cm, 94.2 ± 18.8 kg, 34.9 ± 6.4 kg·m^-2^, 43.8 ± 4.2%) participated in this study. Participants were weight stable and not participating in additional weight loss programs.

**Methods:**

Participants were assigned to either a no exercise + no diet control (CON), a no diet + exercise group (ND), or one of four diet + exercise groups (presented as kcals; % carbohydrate: protein: fat): 1) a high energy, high carbohydrate, low protein diet (HED) [2,600; 55:15:30%], 2) a very low carbohydrate, high protein diet (VLCHP) [1,200 kcals; 63:7:30%], 3) a low carbohydrate, moderate protein diet (LCMP) [1,200 kcals; 50:20:30%] and 4) a high carbohydrate, low protein diet (HCLP) [1,200 kcals; 55:15:30%]. Participants in exercise groups (all but CON) performed a pneumatic resistance-based, circuit training program under supervision three times per week.

**Measurements:**

Anthropometric, body composition, resting energy expenditure (REE), fasting blood samples and muscular fitness assessments were examined at baseline and weeks 2, 10 and 14.

**Results:**

All groups except CON experienced significant reductions (*P *< 0.05 – 0.001) in waist circumference over 14 weeks. VLCHP, LCHP and LPHC participants experienced similar but significant (*P *< 0.05 – 0.001) reductions in body mass when compared to other groups. Delta responses indicated that fat loss after 14 weeks was significantly greatest in VLCHP (95% CI: -5.2, -3.2 kg), LCMP (-4.0, -1.9 kg) and HCLP (-3.8, -2.1 kg) when compared to other groups. Subsequent reductions in % body fat were significantly greater in VLCHP, LCMP and HCLP participants. Initial dieting decreased (*P *< 0.05) relative REE similarly in all groups. All exercise groups significantly (*P *< 0.05) improved in muscular fitness, but these improvements were not different among groups. Favorable but non-significant mean changes occurred in lipid panels, glucose and HOMA-IR. Leptin levels decreased (*P *< 0.05) in all groups, except for CON, after two weeks of dieting and remained lower throughout the 14 week program. Exercise participation resulted in significant improvements in quality of life and body image.

**Conclusion:**

Exercise alone (ND) appears to have minimal impact on measured outcomes with positive outcomes apparent when exercise is combined with a hypoenergetic diet. Greater improvements in waist circumference and body composition occurred when carbohydrate is replaced in the diet with protein. Weight loss in all diet groups (VLCHP, LCMP and HCLP) was primarily fat and stimulated improvements in markers of cardiovascular disease risk, body composition, energy expenditure and psychosocial parameters.

## Introduction

The prevalence of obesity in the United States and throughout the world continues to increase. An estimated 1.2 billion people in the world are overweight with 300 million of them being obese [1,2]. Research over the last several decades indicates that regular activity and appropriate energy intake can play critical roles in preventing and managing the negative health consequences of diabetes, obesity and other cardiovascular diseases [3-7]. Initially, weight loss programs focused on restricting energy intake, but sharp reductions in energy intake have been shown to result in fat-free mass reductions and negatively impact metabolic rate [8]. Replacing dietary carbohydrate with protein without changing fat intake continues to be researched as a potential strategy to improve health and promote weight loss. While replacing dietary protein with fat in isocaloric amounts doesn't appear to improve the loss of body mass [9-11], studies in the last several years have shown that following diets which replace carbohydrate with protein may stimulate greater weight loss [12,13] and improve body composition changes [12,14-16]. Interestingly, findings have also reported that replacing dietary carbohydrate with protein may also improve serum-based markers of diabetes and cardiovascular disease (e.g. fasting insulin, glycemic responses, triglycerides and total cholesterol) [12,14-16]. While many of these studies examined a dietary intervention, several did not include any form of exercise component [14-16]. For those studies that did investigate a combination of dietary intake with and without exercise, some have lacked additional control measures to fully elucidate the impact of these dietary approaches when combined with a prolonged exercise intervention [12,13]. Furthermore, the mode of exercise is an important consideration, and the incorporation of some aspect of resistance-based exercise during a weight loss program may help to maintain fat free mass, sustain resting energy expenditure levels and promote higher levels of function [8]. Currently, the number of investigations that have combined a resistance-based exercise program with a dietary intervention that focuses on replacing carbohydrate with protein is limited [8], making it difficult to adequately determine which form of exercise and dietary intervention has the greatest potential to help people achieve their weight loss and other health-related goals.

Curves International is currently the largest fitness franchise in the world with over 4 million members in 10,000 clubs in over 69 countries [17]. The typical Curves member is a 30 – 60 year old sedentary female who ranges from being slightly overweight to obese according to BMI standards [18]. The Curves program attracts a large segment of women to participate in their program who have many times previously avoided exercising at coed fitness facilities and unsuccessful with prior weight loss efforts [19]. Moreover, rather than incorporating traditional forms of aerobic exercise as the primary mode of physical activity, participants in the Curves program perform bi-directional, pneumatic resistance-training exercises (in a circuit format) interspersed with low-impact callisthenic exercises for 30-minutes, three times per week [19]. In conjunction with the exercise program, several dietary regimens are utilized throughout the program. A high calorie, high carbohydrate, low protein low fat diet (2,600 kcals: 55:15:30% (carbohydrate: protein: fat) is recommended for women who have low resting energy expenditure, defined as <10% below predicted RMR [20], and a low daily energy intakes. Otherwise, a low calorie, low carbohydrate, high protein, low fat diet (1,000 – 1,200 kcals: 20:50:30%) or a low calorie, high carbohydrate, low protein, low fat diet (1,000 – 1,200 kcals: 55:15:30%) is recommended depending on carbohydrate tolerance for an initial diet period of 2 – 4 weeks before increasing caloric intake to 1,600 kcals while maintaining a similar (but not identical) macronutrient distribution until weight loss goals are achieved. Once achieved, weight maintenance is promoted by adhering to a 2,200 – 2,600 kcals·d^-1 ^diet based on American Dietetic Association guidelines (% carbohydrate:protein:fat = 55:15:30) with intermittent periods of reduced caloric intake (e.g. 1,200 kcals·d^-1^) for period of 2 to 3 days. This program was designed as a means to promote and sustain weight loss in women while preserving fat free mass and resting energy expenditure [19].

While previous research provides general conceptual support for this program, the safety and efficacy of the Curves program has yet to be investigated. Therefore, the objectives of this study were two-fold: 1) to examine the efficacy of replacing dietary carbohydrate with protein while completing a weekly resistance-based circuit exercise program and 2) to determine the safety and efficacy of following the Curves fitness and weight loss program [19] over a prolonged period. It was hypothesized that all groups participating in the exercise program, irrespective of dietary assignment, would experience significant improvements in anthropometric measures, body composition, and improve their fitness and risk for cardiovascular disease. Further hypotheses were made that those participants following diets that replaced dietary carbohydrate with protein would experience greater improvements in anthropometrics and body composition in addition to greater improvements in cardiovascular disease markers.

## Methods

### Experimental Approach

**Figure 1:**
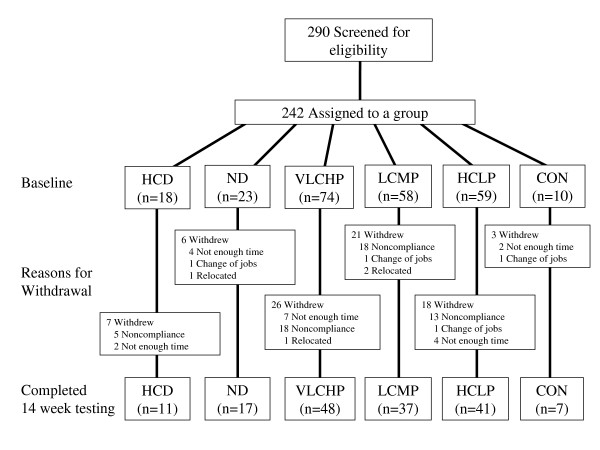
**Standard consort diagram illustrating people who provided interest in participating, those that were screened, how group assignments were made, who completed program and reasons for termination of participation**.

Two primary objectives were studied in this investigation, first to examine the impact of replacing dietary carbohydrate with dietary protein to varying degrees and second to assess the effectiveness of following the exercise and diet recommendations of the Curves program that were published at the time this study was initiated [19]. Participants were placed into one of six groups: no diet + no exercise control (CON), no diet + exercise (ND), high carbohydrate, high energy diet (HED) + exercise (2,600; 55:15:30%); very low carbohydrate, high protein (VLCHP) + exercise (1,200; 63:7:30%), low carbohydrate, moderate protein (LCMP) + exercise (1,200: 50:20:30%), high carbohydrate, low protein (HCLP) + exercise group (1,200: 55:15:30%). All participants were tested over the course of a 14 week period for changes in anthropometrics, body composition, cardiovascular and muscular fitness, serum and whole clinical and hormonal changes and psychosocial parameters after 0, 2, 10, and 14 weeks of following their assigned diet and exercise program. Primary outcomes in this study were identified as waist circumference while all secondary outcomes were body mass and DXA body composition parameters. Finally, tertiary outcomes were associated with changes in resting energy expenditure, cardiovascular and muscular fitness and all serum and whole blood safety and hormonal markers. Testing after 2 weeks was completed to assess the acute changes in energy expenditure and hormonal status after the most energy-restrictive phase of the diets (1,200 kcals·d^-1^) while testing at 10 and 14 weeks provide for follow-up assessments after less-restrictive phases of the dieting program. Participation in the exercise program was hypothesized to promote greater improvements in all primary and secondary outcomes measures when compared to the non-exercise controls (CON). A standard consort diagram is available which outlines the number of participants who were screened (n = 290), assigned to a group (n = 242), including how many to each intervention group and those that completed the entire 14 week protocol (n = 161) (see Figure 1).

### Participants

One-hundred sixty one sedentary, obese, female participants (38.5 ± 8.5 yrs, 164.2 ± 6.7 cm, 94.2 ± 18.8 kg, 34.9 ± 6.4 kg·m^-2^, 43.8 ± 4.2%) participated in this study (Table 1). Participants were not allowed to participate in this study if at baseline they reported: 1.) being currently diagnosed with any metabolic or cardiovascular disorder including known electrolyte abnormalities; heart disease, arrhythmias, diabetes, thyroid disease, hypogonadism; or a history of hypertension, hepatorenal, musculoskeletal, autoimmune, or neurologic disease; 2.) any current prescriptions for thyroid, hyperlipidemic, hypoglycemic, anti-hypertensive, or androgenic medications; 3.) taking any ergogenic levels of nutritional supplements that may affect muscle mass (e.g. creatine, β-hydroxy-β-methylbutyrate [HMB]), dehydroepiandrosterone [DHEA], or weight loss (e.g. ephedra, thermogenics, etc) within one year prior to the starting the study; 4.) any condition which classified them as high risk for cardiovascular disease according to American College of Sports Medicine criteria [21]; 5) participating in any other form of a diet or exercise program during the 12 months prior to starting the study. Participants meeting eligibility criteria were informed of the requirements of the study and signed informed consent statements in compliance with the Human Subjects Guidelines of Baylor University and the American College of Sports Medicine.

**Table 1:** Baseline anthropometric, dietary analysis, body composition and biochemical parameters for the high energy, high carbohydrate diet + exercise (HED; 2,600: 55:15:30), no diet + exercise (ND), very low carbohydrate, high protein diet + exercise (VLCHP: 1,200; 63:7:30), low carbohydrate, moderate protein diet + exercise (LCMP: 1,200; 50:20:30), high carbohydrate, low protein diet + exercise (HCLP: 1,200; 55:15:30) and no diet + no exercise control (CON).

*Demographics*	Grand Mean	HED	ND	VLCHP	LCMP	HCLP	CON	*P*-value
Age (years)	38 ± 9	35 ± 10	37 ± 10	39 ± 7	38 ± 9	41 ± 8	32 ± 10	0.10
Height (cm)	164 ± 7	164 ± 6	163 ± 8	165 ± 6	165 ± 7	163 ± 7	163 ± 6	0.65
Weight (kg)	94 ± 19	93 ± 13	81 ± 13^d^	108 ± 19	92 ± 18^d^	87 ± 13^d^	89 ± 18	<0.001
Body mass index	35 ± 6	34 ± 4	31 ± 4^d^	39 ± 7	34 ± 6^d^	33 ± 4^d^	34 ± 6	<0.001
								
(kg·m^-2^)								
								
Waist (cm)	101 ± 13	104 ± 7	93 ± 12^d^	108 ± 14	101 ± 13	96 ± 11^d^	95 ± 13	<0.001
DXA fat-free mass (kg)	49 ± 8	49 ± 7	43 ± 7^d^	54 ± 7	48 ± 8^d^	45 ± 6^d^	48 ± 6	<0.001
DXA fat mass (kg)	39 ± 11	37 ± 7	32 ± 7^d^	46 ± 12	37 ± 10^d^	35 ± 8^d^	38 ± 9	<0.001
								
REE (kcals·d^-1^)	1730 ± 300	1331 ± 193	1595 ± 228^d, f^	1955 ± 278^f^	1705 ± 249^d, f^	1645 ± 222^d, f^	1753 ± 274^f^	<0.001
								
Systolic blood pressure (mm Hg)	125 ± 13	127 ± 20	119 ± 10	129 ± 15	124 ± 9	126 ± 10	121 ± 14	0.10
Diastolic blood pressure (mm Hg)	83 ± 8	86 ± 14	79 ± 7	84 ± 8	83 ± 8	84 ± 7	79 ± 7	0.13
VO_2 _(ml/kg/min)	21 ± 5	22 ± 3	24 ± 5	19 ± 6	22 ± 5	22 ± 4	23 ± 5	<0.001
								
*Biochemical parameters*	Grand Mean	HED	ND	VLCHP	LCMP	HCLP	CON	*P*-value

Total cholesterol	5.0 ± 0.9	4.9 ± 0.8	5.1 ± 0.9	4.9 ± 0.7	5.0 ± 0.9	5.1 ± 1.1	5.3 ± 1.1	0.87
(mmol·L^-1^)								
								
HDL cholesterol	1.4 ± 0.3	1.3 ± 0.2	1.4 ± 0.3	1.3 ± 0.3	1.4 ± 0.3	1.4 ± 0.2	1.3 ± 0.3	0.36
(mmol·L^-1^)								
								
LDL cholesterol	3.0 ± 0.8	3.1 ± 0.7	3.1 ± 0.8	3.0 ± 0.6	2.8 ± 0.8	3.0 ± 1.0	3.3 ± 0.9	0.62
(mmol·L^-1^)								
								
Triglycerides	1.5 ± 0.9	1.0 ± 0.4	1.3 ± 0.5	1.6 ± 1.1	1.5 ± 0.9	1.5 ± 0.7	1.6 ± 0.8	0.50
(mmol·L^-1^)								
								
Glucose	5.3 ± 0.7	5.1 ± 0.5	5.0 ± 0.3	5.3 ± 0.7	5.3 ± 0.9	5.3 ± 0.5	5.2 ± 0.7	0.57
(mmol·L^-1^)								
								
Insulin	4.9 ± 4.0	3.3 ± 1.6	4.3 ± 2.3	6.0 ± 3.9	5.9 ± 6.0	3.8 ± 2.4	3.7 ± 2.0	<0.05
(pmol·L^-1^)								
								
HOMA-IR	1.15 ± 1.0	0.75 ± 0.4	0.95 ± 0.5	1.43 ± 1.0	1.40 ± 1.4	0.89 ± 0.6	0.89 ± 0.6	<0.05
								
Leptin	103 ± 48	98 ± 39	94 ± 35	107 ± 37	113 ± 67	99 ± 48	84 ± 39	0.56
(pgm·L^-1^)								
								
*Dietary Intake*	Grand Mean	HED	ND	VLCHP	LCMP	HCLP	CON	*P*-value

Caloric intake (kcal/kg/day)	22.4 ± 6.9	23.7 ± 4.2	21.2 ± 6.6	20.5 ± 7.5	23.3 ± 7.5	23.6 ± 6.0	---	0.29
Carbohydrate (g/kg/day)	2.8 ± 1.1	3.0 ± 0.8	2.7 ± 0.8	2.4 ± 1.4	2.8 ± 1.1	3.1 ± 1.0	---	0.19
Protein (g/kg/day)	0.88 ± 0.3	1.0 ± 0.2	0.8 ± 0.2	0.9 ± 0.4	0.9 ± 0.5	0.8 ± 0.2	---	0.76
Fat (g/kg/day)	0.89 ± 0.3	0.9 ± 0.2	0.8 ± 0.4	0.9 ± 0.4	0.9 ± 0.4	0.9 ± 0.3	---	0.90

All data is presented as means ± SD at baseline. Significance level was set at 0.05.

^d^Different than VLCHP, *P *< 0.001 – 0.05; ^f^Different than HED, *P *< 0.001. NOTE: No other differences at baseline existed (*P *> 0.05).

### Testing Sessions

Recruitment occurred by flyers posted in local newspapers, campus flyers and television announcements. Interested participants first contacted the laboratory for pre-screening before completing a familiarization session where they were additionally screened and provided detailed information about the exercise program, diet and testing protocols. All participants completed informed consent documents during this familiarizations session. Prior to all testing sessions including baseline, participants completed a 4 d dietary record, observed an 8 h fast and refrained from vigorous physical activity for 24 h prior to each testing session. All testing sessions were scheduled at similar times in the morning to control for diurnal variations. All baseline, 10 week and 14 week testing sessions, respectively, were identical in nature and consisted of blood collection (blood lipids, metabolic panels), anthropometric assessments (body mass, body mass index, waist circumference), resting energy expenditure, muscular fitness and cardiorespiratory assessments, body composition analysis (DXA) and psychosocial assessments (e.g. short-form-36 (SF-36) quality of life, social physique anxiety scale (SPA), Rosenberg self-esteem scale (RSE), Cash Body Image questionnaire). An additional testing session occurred after 2 weeks of dieting and consisted of all baseline measures with the exception of cardiorespiratory and muscular fitness assessments. Upon completion of baseline testing, all participants, with the exception of the no exercise control group (CON), began a thrice weekly 30 min pneumatic circuit-style resistance-based circuit exercise program interspersed with callisthenic activities. During each workout, fitness supervisors provided exercise instruction and assisted with self-monitoring of heart rate to maintain an exercise heart rate between 60–80% target heart rate using age-predicted maximal heart rate (220 – age) and the Karvonen method.

### Diet Assignments

Upon completion of baseline testing, participants were matched according to body mass and age into either a no diet + no exercise control (CON), a no diet + exercise group (ND), or one of four dietary regimens. The diets followed the Curves diet recommendations at the time this study was conducted [19] and consisted of replacing dietary carbohydrate with dietary protein while keeping dietary fat intake standardized at 30% of total daily caloric intake [14]. Participant's whose measured REE was less than 90% of predicted REE (no activity factor was incorporated) using the Harris-Benedict prediction equation [22] were categorized as hypo-metabolic [23]. They were assigned to follow a 2,600 kcals·d^1 ^at a macronutrient ratio of 55:15:30% (carbohydrate:protein:fat), high-energy, high carbohydrate diet (HED) for 14 weeks to assess anthropometric and weight loss changes. A brief questionnaire was administered to all participants at familiarization to provide a qualitative indication of carbohydrate/glycemic tolerance, which is used by the Curves program to assist with dietary assignment and thus was adopted as part of this investigation [19]. Recent studies by our group have illustrated that in a large group of overweight and obese women this questionnaire effectively associates responses with indicators of metabolic syndrome [24,25]. Those participants responding in a positive fashion (e.g. provided answers indicative of carbohydrate intolerance) were assigned to either the VLCHP or LCMP groups. Participants responding negatively were assigned to the HCLP diet [19]. In each of these diets, participants ingested 1,200 kcals·d^-1 ^for 2-weeks (phase 1). Over the next 8-weeks, energy intake was increased to 1,600 kcals·d^-1 ^(phase 2) providing for a weight loss period of 10 weeks consisting of caloric intakes ranging from 1,200 – 1,600 kcals·d^-1^. The remaining 4 weeks (phase 3) utilized a weight maintenance approach in which participants followed a 2,600 kcals·d^-1 ^using the recommend macronutrient breakdown (55:15:30%; carbohydrate: protein: fat) by the American Dietetic Association with intermittent 2 – 3 day periods of phase 1 dieting. Similar weight maintenance models have been previously investigated [14] for their effectiveness and this approach is utilized as a weight maintenance approach during this program. To facilitate adherence and comprehension of each dietary assignment, a team of reregistered dieticians performed an additional diet familiarization session with each participant and developed customized menu booklets, providing sample diets and food substitutions for each diet phase. The booklets were used as checklists upon which all study participants were required to return after completing the study. Food logs were maintained for 4 days prior to each testing session. Registered dietitians reviewed the dietary records with the participant to promote compliance to the diet programs.

### Procedures

#### Dietary Inventories

Prior to each testing session, participants recorded all food and fluid intake over a 4-d period (including one weekend day), which was reflective of their normal dietary intake. Dietary inventories were then reviewed by a registered dietician and analyzed for average caloric and macronutrient intake using ESHA Food Processor (Version 8.6) Nutritional Analysis software (Salem, OR).

#### Anthropometrics

Each testing session, height and body mass were determined according to standard procedures using a Healthometer (Bridgeview, IL) self-calibrating digital scale with an accuracy of ± 0.02 kg. Waist circumference was measured using a Golnick tensiometer using standard ACSM criteria [21]. Resting heart rate was measured via palpation of the radial artery and resting blood pressure was determined using a mercury sphygomometer (American Diagnostic Corporation, model # AD-720, Hauppuage, NY) according to previously accepted procedures [21].

#### Body Composition and Energy Expenditure Assessments

Resting energy expenditure (REE) assessments were made using a Parvo Medics TrueMax 2400 Metabolic Measurement System (Sandy, UT). This test was a non-exertional test performed in a fasted state with the participants lying supine on an exam table. A clear, hard plastic hood and soft, clear plastic drape was placed over the participants' neck and head in order to determine resting oxygen uptake and energy expenditure. All participants remained motionless without falling asleep for approximately 20 minutes. Data were recorded after the first ten minutes of testing during a five minute period of time in which criterion variables (e.g. VO_2 _[Lmin^-1^]) changed less than 5% [26]. Using a sub-sample of 14 participants from this investigation, test-retest correlations (*r*) of collected VO_2 _in L·min^1 ^ranged from 0.315 – 0.901 (: 0.638) and coefficient of variation ranged from 8.2% – 12.0% (: 9.9%) with a mean intra-class coefficient of 0.942, p < 0.001. Participants then had their bone density and body composition assessed with a whole-body scan using a Hologic QDR-4500W DXA using software version 9.8 (Waltham, MA). Mean coefficients of variation in bone mineral content and bone mineral density measurements on the spine phantom ranged between 0.41 – 0.55%. Test-retest reliability studies performed on male athletes with this DXA machine have previously yielded mean coefficients of variation for total bone mineral content and total fat free/soft tissue mass of 0.31 – 0.45% with a mean intra-class correlation of 0.985 [27].

#### Cardiopulmonary Exercise Tests

At baseline and after 10 and 14 weeks, participants completed a peak cardiopulmonary exercise test according to the Bruce protocol [28]. Using standard electrode placement and a Quinton 710 ECG unit (Bothell, Washington, USA), 12-lead electrocardiogram tests were also made to assess heart function according to previously established criteria [21]. An exercise heart rate of 85% of predicted maximal heart rate was criteria used to standardized exertion during each test. Standard ACSM test termination criteria were monitored and followed throughout each test [21]. Resting and exercise expired gases were collected using a Parvo Medics TrueMax 2400 Metabolic Measurement System (Sandy, Utah, USA). Calibration of gas and flow sensors was completed every morning prior to testing and was found to be within 3% of the previous calibration point.

#### Maximal Strength and Endurance Assessments

At baseline and after 10 and 14 weeks, participants had their one-repetition maximum (1 RM) determined using the bench press and leg press exercises for changes in maximal strength. Muscular endurance was assessed by having participants perform as many repetitions as possible with 80% of their pre-determined 1 RM. Standard lifting techniques and criteria according to National Strength and Conditioning Association (NSCA) were followed throughout all testing [29]. Test to test reliability of performing these strength tests in our lab on resistance-trained participants have yielded low mean coefficients of variation and high reliability for the bench press (1.9%, intra-class r = 0.94) and hip sled/leg press (0.7%, intra-class r = 0.91).

#### Blood Collection Procedures

Fasted whole blood and serum samples were collected using standard phlebotomy techniques. Whole blood samples were analyzed for complete blood counts with platelet differentials using an Abbott Cell Dyn 3500 (Abbott Laboratories, Abbott Park, Illinois, USA) automated hematology analyzer. Serum samples were analyzed for a complete metabolic and thyroid panel including ketone assessment of beta-hydroxybutyrate using a calibrated Dade Behring Dimension RXL (Deerfield, Illinois, USA) automated clinical chemistry analyzer. Coefficient of variation for the tests using this analyzer was similar to previously published data for these tests (range: 1.0 to 9.6%) [30]. Remaining serum was assayed using standard commercially available (DS Laboratories, Webster, Texas, USA) enzyme-linked immunoabsorbent assays (ELISAs) for leptin and insulin. Serum concentrations were assessed in duplicate using a Wallac-Victor IV (Perkin-Elmer Life Sciences, Boston, Massachusetts, USA) micro plate reader at an optical density of 450 nm against a known standard curve using standard ELISA procedures. Intra- and inter-assay CVs of 5.4 – 6.9 and 3.8 – 7.3% at ~5.0 – 21.1 ng·mL^-1 ^existed for leptin and 1.3 – 2.6 and 5.2 – 6.2% at ~7.84 – 44.23 μIU·ml^-1 ^for insulin, respectively.

#### Psychosocial Assessments

Prior to any testing, participants completed the SF-36 Health-Related Quality of life (QOL) inventory [31], social physique anxiety scale (SPA) [32], Rosenburg self-esteem scale (RSE) [33], and a Cash body image questionnaire [34].

#### Exercise Program

With the exception of the CON group, all participants participated in a supervised Curves exercise program three days per week throughout the fourteen week protocol (a total of 42 workouts). Each circuit-style workout consisted of 14 exercises (e.g. elbow flexion/extension, knee flexion/extension, shoulder press/lat pull, hip abductor/adductor, chest press/seated row, horizontal leg press, squat, abdominal crunch/back extension, pec deck, oblique, shoulder shrug/dip, hip extension, side bends and stepping) constructed with pneumatic or hydraulic resistance that targeted opposing muscle groups in a concentric-only fashion. Participants were informed of proper use of all equipment and were instructed to complete as many repetitions in a 30 s time period. In a continuous, interval fashion, participants performed floor-based callisthenic (e.g. running/skipping in place, arm circles, etc.) exercises for a 30 s time period after each resistance exercise in an effort to maintain a consistent exercise heart rate that corresponded to 60% to 80% of their maximum heart rate [21]. All workouts were supervised by trained fitness instructors who assisted with proper exercise technique and maintenance of adequate exercise intensity. Participants were required to complete two complete circuits which corresponded to exercising for approximately 28 minutes followed by a standardized whole-body stretching routine. Compliance to the exercise program was set a priori at a minimum of 70% compliance (30/42 exercise sessions). Participants were allowed to add an additional workout on a non-consecutive day to assist with maintaining appropriate exercise compliance and in rare instances (n<5), an additional week was added to the intervention.

#### Statistical Analysis

All data are presented as means and 95% CI for all variables for the CON, ND, HED, VLCHP, LCMP and HCLP groups, respectively. All nutritional intake data was normalized to kilograms of body mass and analyzed using 5 × 4 (without ND group) repeated measures ANOVA. Eleven participants completed all training and testing, but did not turn in accurate food records. Consequently, all statistical analysis of nutritional intake was made on the remaining 143 participants from the original dataset as individuals in the CON group (n = 7) completed dietary analysis at weeks 0 and 14 and were not included in the statistical analysis. Resting energy expenditure was analyzed using a 6 × 4 (group × test [0, 2, 10 and 14 weeks]) ANCOVA for baseline body mass. All remaining data were analyzed using 6 × 3 (group × test [0, 10 and 14 weeks]) repeated measures ANOVA. 14-week change from baseline (delta) values were calculated and a mean with 95% confidence interval was instructed to assess changes over time. When significant group × time interaction effects were found, factor analysis of the main effects was determined using pairwise comparisons and one-way ANOVA when appropriate. Select Pearson correlations were calculated between the psychosocial and body composition variables to identify any relationships between these groups of data. Percent change from baseline was calculated and reported to evaluate any changes in this data. Post-hoc power analysis all primary and secondary outcomes (e.g., waist circumference, body mass, body composition via DXA) revealed observed powers values ranging from 0.821 – 0.998 with partial eta-squared values ranging from 0.062 – 0.115. All statistical decisions were made using an alpha level of 0.05. Individual responses are plotted for waist circumference, body mass, DEXA fat mass and triglyceride changes while groups mean are graphed for changes in absolute REE with the mean and 95% confidence interval offset next to each figure.

## Results

### Nutritional Intake

With the exception of the control group (CON) and the exercise only group (ND), all participants were provided a diet that provided the desired percentage of carbohydrate, protein, fat and energy intake. Participants completed 4 day dietary recalls after 0, 2, 10 and 14 weeks of following their assigned diet and the exercise program. Results show that participants in the diet groups reduced their energy intake throughout the program (Time effect: *P *< 0.001; G × T; *P *= 0.10) and altered carbohydrate (G × T; *P *= 0.001), protein (G × T; *P *= 0.01) and fat intake (G × T; *P *= 0.04) as intended. Mean values of energy intake, however, revealed that while participants did alter their diets and followed the designed patterns, the anticipated caloric intake for some groups (i.e., particularly the HED group) did not achieve the intended levels, but this did not influence the study's ability to investigate the impact of altering macronutrient ratios in a diet while exercising.

### Anthropometrics and Body Mass

**Figure 2:**
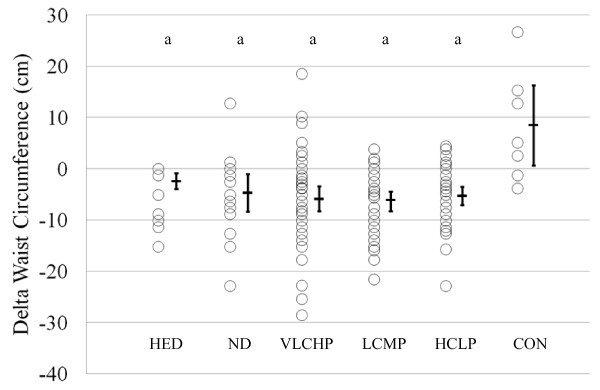
**Delta change in waist circumference (cm) at 14 weeks**. Data are presented as individual changes from baseline. Each respective individual group mean and 95% confidence interval are placed immediately to the right of each data group. HED = high-energy, high carbohydrate diet + exercise (n = 11); ND = no diet + exercise (n = 17); VLCHP = very low carbohydrate, high protein diet + exercise (n = 48); LCMP = Low carbohydrate, moderate protein + exercise (n = 37); HCLP = High carbohydrate, low protein + exercise (n = 41); CON = no diet + no exercise (n = 7). ^a^Different than CON (p < 0.05).

**Figure 3:**
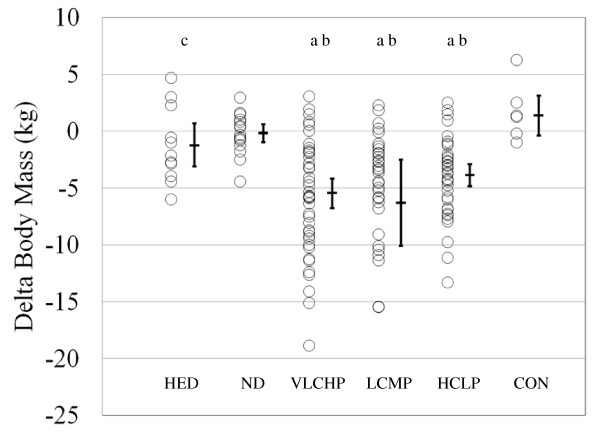
**Delta change in body mass (kg) at 14 weeks**. Data are presented as individual changes from baseline. Each respective individual group mean and 95% confidence interval are placed immediately to the right of each data group. HED = high-energy, high carbohydrate diet + exercise (n = 11); ND = no diet + exercise (n = 17); VLCHP = very low carbohydrate, high protein diet + exercise (n = 48); LCMP = Low carbohydrate, moderate protein + exercise (n = 37); HCLP = High carbohydrate, low protein + exercise (n = 41); CON = no diet + no exercise (n = 7). ^a^Different than CON, *P *< 0.05. ^b^Different than ND+E, *P *< 0.05. ^c^Different than LCMP, *P *< 0.05.

Waist circumference decreased in the HED, VLCHP and LCMP after 14 weeks (P < 0.001 for all groups, respectively). After 14 weeks, significant time effects for waist circumference were found in all groups except CON (Table 2). As expected, when comparing all groups who exercised against those who did not exercise (CON), a significant group × time interaction was found for changes in waist circumference (Figure 2; *P *< 0.05). After 10 weeks, those participants who restricted caloric intake and exercised experienced a significantly greater loss of body mass than HED, ND and CON (*P *< 0.01; Figure 3). Participants following the VLCHP diet experienced significantly greater body mass loss in comparison to the ND (*P *< 0.005) and CON (*P *< 0.005) diets. Similar findings were found for those individuals following the LCMP diet when compared to the ND (*P *< 0.005) and CON (*P *< 0.05) groups. Loss of body mass for the HCLP approached significance when compared to the ND (*P *= 0.077) and CON (*P *= 0.095) groups, but were subsequently not significant. Furthermore, all participants maintained their body mass over the last four weeks of the investigation (Table 2; *P *= 0.63).

**Table 2:** Anthropometric, body mass, body composition and energy expenditure changes for the high energy, high carbohydrate diet + exercise (HED; 2,600: 55:15:30), no diet + exercise (ND), very low carbohydrate, high protein diet + exercise (VLCHP: 1,200; 63:7:30), low carbohydrate, moderate protein diet + exercise (LCMP: 1,200; 50:20:30), high carbohydrate, low protein diet + exercise (HCLP: 1,200; 55:15:30).

				*P*-value	
				
Variable	Group	Mean	14 Week Delta	Within Group	G × T
Waist (cm)	HED	-2.8	(-4.3, -1.2)^a^	<0.001	<0.05
	ND	-5.1	(-8.8, -1.4)^a^	<0.05	
	VLCHP	-6.3	(-8.7, -3.8)^a^	<0.001	
	LCMP	-6.7	(-8.7, -4.8)^a^	<0.001	
	HCLP	-5.7	(-7.5, -3.9)^a^	<0.05	
	CON	8.2	(+0.3, +16.1)	0.16	

Body Mass (kg)	HED	-1.3	(-3.2, +0.7)^c^	0.26	<0.001
	ND	-0.2	(-1.0, +0.6)	0.51	
	VLCHP	-5.6	(- 7.0, -4.3)^a, b^	<0.001	
	LCMP	-6.5	(-10.4, -2.6)^a, b^	<0.01	
	HCLP	-4.0	(-5.0, -3.0)^a, b^	<0.001	
	CON	1.4	(-0.4, +3.2)	0.28	

DXA Fat-Free Mass (kg)	HED	-0.1	(-0.7, +0.4)	0.36	<0.05
	ND	0.1	(-0.6, +0.7)	0.81	
	VLCHP	-1.3	(-1.8, -0.7)	<0.001	
	LCMP	-1.1	(-1.6, -0.6)	<0.001	
	HCLP	-0.6	(-1.1, -0.1)	<0.001	
	CON	0.5	(-0.8, +1.8)	0.62	

DXA Fat Mass (kg)	HED	-0.9	(-2.7, +0.9)^d^	0.38	<0.001
	ND	-0.8	(-1.6, +0.1)	0.09	
	VLCHP	-4.2	(-5.2, -3.2)^a, b^	<0.001	
	LCMP	-2.9	(-4.0, -1.9)^a, b^	<0.001	
	HCLP	-2.9	(-3.8, -2.1)^a, b^	<0.001	
	CON	0.3	(-1.8, +2.4)	0.85	

DXA % Fat (%)	HED	-0.6	(-1.8, +0.6)	0.42	0.21
	ND	-0.6	(-1.3+0.2)	0.19	
	VLCHP	-2.0	(-2.7, -1.3)	<0.005	
	LCMP	-1.7	(-2.4, -0.9)	<0.001	
	HCLP	-2.0	(-2.6, -1.4)	<0.001	
	CON	0.0	(-1.5, +1.5)	0.96	

REE (kcals·d^-1^)	HED	413	(+264, +562)	<0.001	0.45
	ND	83	(-5, +135)^e^	0.08	
	VLCHP	-37	(-84, +42)^e^	0.06	
	LCMP	18	(-58, +94)^e^	0.52	
	HCLP	90	(+22, +160)^d, e^	<0.05	
	CON	-30	(-105, +63)^e^	0.91	

Dietary intake is presented as energy (i.e. calories) intake: % carbohydrate: protein: fat. All data is presented as the mean plus the 95% confidence intervals for the delta response at week 14. Main effects for time are provided as within-group *P*-values. Group × time interaction effects are provided as GxT *P*-values. Significance level was set at 0.05.

^a^Different than CON, *P *< 0.05, ^b^Different from ND, *P *< 0.05, ^c^Different from LCMP, *P *< 0.05, ^d^Different than VLCHP, *P *< 0.05, ^e^Different from HED, *P *< 0.05.

### Body Composition

**Figure 4:**
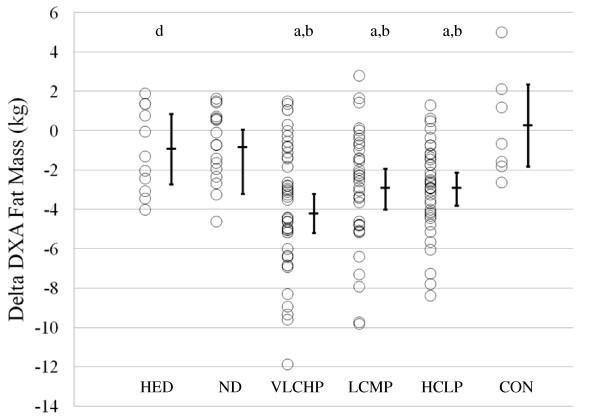
**Delta change in DXA fat mass (kg) at 14 weeks**. Data are presented as individual changes from baseline. Each respective individual group mean and 95% confidence interval are placed immediately to the right of each data group. HED = high-energy, high carbohydrate diet + exercise (n = 11); ND = no diet + exercise (n = 17); VLCHP = very low carbohydrate, high protein diet + exercise (n = 48); LCMP = Low carbohydrate, moderate protein + exercise (n = 37); HCLP = High carbohydrate, low protein + exercise (n = 41); CON = no diet + no exercise (n = 7). ^a^Different than CON, *P *< 0.05. ^b^Different than ND, *P *< 0.05. ^d^Different than VLCHP, *P *< 0.05.

Body composition was assessed at 0, 10 and 14 weeks to determine changes in fat-free and fat mass (Table 2). All groups that restricted caloric intake and exercised (VLCHP, LCMP, HCLP) experienced significant but similar within group reductions over time in DXA fat-free mass. Similar changes in DXA fat mass (Figure 4) were found in VLCHP, LCMP and HCLP after both 10 weeks and 14 weeks which resulted in an overall reduction of DXA % body fat in the VLCHP, LCMP and HCLP groups at both 10 weeks and 14 weeks (Table 2). DXA fat reductions in the VLCHP group were significantly greater after 14 weeks when compared to HED, ND and CON (*P *< 0.05).

### Energy Expenditure

**Figure 5:**
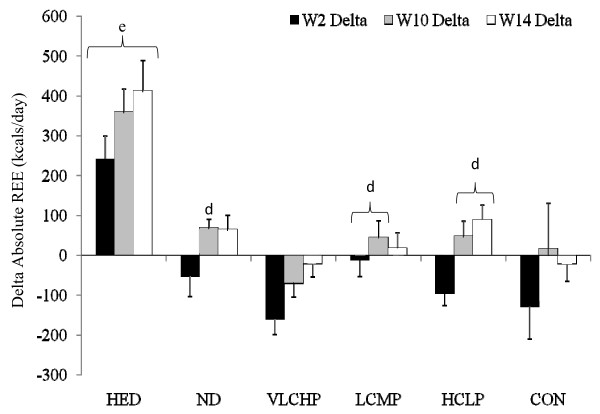
**Delta change in absolute resting energy expenditure (kcal·d^-1^) at 14 weeks**. Each respective individual group mean and 95% confidence interval are placed immediately to the right of each data group. HED = high-energy, high carbohydrate diet + exercise (n = 11); ND = no diet + exercise (n = 17); VLCHP = very low carbohydrate, high protein diet + exercise (n = 48); LCMP = Low carbohydrate, moderate protein + exercise (n = 37); HCLP = High carbohydrate, low protein + exercise (n = 41); CON = no diet + no exercise (n = 7). ^d^Different from VLCHP, *P *< 0.05. ^e^Different from all other groups, *P *< 0.05.

Fasting resting energy expenditure (REE) measurements were obtained at 0, 2, 10 and 14 weeks (not all data is shown) to determine changes in resting energy expenditure (Figure 5 and Table 2). To control for the influence of baseline body mass on REE, analysis of the absolute resting energy expenditure data was performed using an ANCOVA and revealed no overall significant group × time interaction effect (*P *= 0.45) although significant within-group increases occurred over time for the HED, ND and HCLP groups. As expected due to the relationship between energy intake and expenditure, oneway ANOVA revealed significantly greater increases from baseline after 2, 10 and 14 weeks (*P *< 0.001 at all three time points) in absolute REE for the HED group when compared to all other groups (Figure 5 and Table 2).

### Cardiovascular and Muscular Fitness Changes

At baseline and after 14 weeks of following the diet and exercise programs, all participants completed maximal strength and muscular endurance assessments (Table 3). As expected, exercise training significantly increased relative peak oxygen uptake in VLCHP (*P *< 0.001), LCMP (*P *< 0.01) and HCLP (*P *< 0.001) while mean reductions in resting heart rate (-3.3 ± 16.5%; *P *= 0.01), systolic blood pressure (-2.8 ± 12.5%; *P *= 0.02), mean arterial pressure (-3.4 ± 10%) and rate pressure product (-5.8 ± 20%) occurred in these groups (data not shown). No significant group × time interaction effect was found for bench press (*P *= 0.44) or leg press 1 RM (*P *= 0.38), although those groups that participated in the exercise program did achieve significant increases (*P *< 0.05–0.001) in relative bench press and leg press 1 RM while CON did not experience changes in either bench press (*P *= 0.59) or leg press 1 RM (*P *= 0.54), respectively. No significant differences were observed among those groups that exercised for 14 weeks suggesting that all dietary regimens equally impacted adaptations to exercise training.

**Table 3:** Cardiorespiratory and muscular fitness changes for the high energy, high carbohydrate diet + exercise (HED; 2,600: 55:15:30), no diet + exercise (ND), very low carbohydrate, high protein diet + exercise (VLCHP: 1,200; 63:7:30), low carbohydrate, moderate protein diet + exercise (LCMP: 1,200; 50:20:30), high carbohydrate, low protein diet + exercise (HCLP: 1,200; 55:15:30).

				*P*-value	
				
Variable	Group	Mean	14 Week Delta	Within Group	G × T
Max VO_2 _(ml·kg·min-^-1^)	HED	1.0	(-0.79, +2.73)	0.48	<0.05
	ND	0.8	(-1.45, +3.00)	0.27	
	VLCHP	3.6	(+2.30, +4.94)	<0.001	
	LCMP	1.1	(+0.13, +2.26)^d^	<0.01	
	HCLP	0.9	(+0.05, +1.67)^d^	<0.001	
	CON	0.1	(-2.92, +3.13)	0.52	

BP 1 RM (kg·kg^-1^)	HED	0.06	(+0.03, +0.09)	<0.01	0.44
	ND	0.06	(+0.04, +0.08)	<0.001	
	VLCHP	0.04	(+0.03, +0.06)	<0.001	
	LCMP	0.09	(+0.03, +0.16)	<0.05	
	HCLP	0.05	(+0.03, +0.06)	<0.001	
	CON	0.02	(-0.02, +0.05)	0.59	

BP Lifting Volume (kg·kg^-1^)	HED	1.8	(-6.07, +9.70)	0.20	0.35
	ND	1.2	(-3.99, +6.37)	0.90	
	VLCHP	3.4	(-0.29, +7.09)	0.30	
	LCMP	1.0	(-3.16, +5.25)	0.18	
	HCLP	2.9	(+0.31, +5.58)	<0.05	
	CON	2.7	(-2.46, +7.81)	0.59	

LP 1 RM (kg·kg^-1^)	HED	0.2	(-0.02, +0.47)	0.11	0.38
	ND	0.2	(+0.09, +0.43)	<0.001	
	VLCHP	0.3	(+0.22, +0.48)	<0.001	
	LCMP	0.5	(+0.20, +0.73)	<0.005	
	HCLP	0.2	(+0.13, +0.32)	<0.001	
	CON	0.1	(-0.15, +0.37)	0.54	

LP Lifting Volume (kg·kg^-1^)	HED	2.0	(-5.76, +9.84)	0.45	0.32
	ND	0.9	(-4.32, +6.08)	0.32	
	VLCHP	4.5	(+0.93, +8.03)	<0.05	
	LCMP	3.9	(-0.79, +8.58)	0.24	
	HCLP	3.9	(+1.40, +6.48)	<0.01	
	CON	2.3	(-2.73, +7.38)	0.28	

Dietary intake is presented as energy (i.e., calories) intake: % carbohydrate: protein: fat. All data is presented as the mean plus the 95% confidence intervals for the delta response at week 14. Main effects for time are provided as within-group P-values. Group × time interaction effects are provided as GxT P-values.Significance level was set at 0.05.

^d^Different than VLCHP, *P *< 0.05

### Lipid Panels

Lipid panels (e.g. triglycerides, total cholesterol, LDL and HDL cholesterol) were completed at weeks 0 and 14 (Table 4). No significant interactive effects for total cholesterol (*P *= 0.90), triglycerides (*P *= 0.31), HDL cholesterol (*P *= 0.78), and LDL cholesterol (*P *= 0.54) values were found in any group. Significant reductions in total cholesterol were found after 14 weeks for found for the VLCHP and HCLP groups. Furthermore, significant but similar within group increases in HDL cholesterol were seen for VLCHP, LCMP and HCLP, but not the HED, ND or CON groups.

**Table 4:** Lipid panel, glucose, insulin, HOMA and leptin changes for the high energy, high carbohydrate diet + exercise (HED; 2,600: 55:15:30), no diet + exercise (ND), very low carbohydrate, high protein diet + exercise (VLCHP: 1,200; 63:7:30), low carbohydrate, moderate protein diet + exercise (LCMP: 1,200; 50:20:30), high carbohydrate, low protein diet + exercise (HCLP: 1,200; 55:15:30).

				*P*-value	
				
Variable	Group	Mean	14 Week Delta	Within Group	G × T
Total Cholesterol (mmo·L^-1^)	HED	-0.14	(-0.49, +0.22)	0.46	0.90
	ND	-0.23	(-0.63, +0.17)	0.28	
	VLCHP	-0.04	(-0.19, +0.11)	0.60	
	LCMP	-0.13	(-0.36, +0.11)	0.31	
	HCLP	-0.17	(-0.33, -0.01)	<0.05	
	CON	-0.15	(-0.62, +0.32)	0.55	

HDL Cholesterol (mmol·L^-1^)	HED	0.05	(-0.04, +0.14)	0.28	0.78
	ND	-0.05	(-0.16, +0.06)	0.40	
	VLCHP	0.02	(-0.03, +0.08)	0.38	
	LCMP	0.01	(-0.05, +0.07)	0.75	
	HCLP	-0.01	(-0.07, +0.05)	0.67	
	CON	0.02	(-0.14, +0.18)	0.80	

LDL Cholesterol (mmol·L^-1^)	HED	-0.28	(-0.64, +0.07)	0.15	0.54
	ND	-0.17	(-0.50, +0.17)	0.35	
	VLCHP	-0.07	(-0.23, +0.09)	0.39	
	LCMP	0.05	(-0.21, +0.31)	0.71	
	HCLP	-0.10	(-0.27, +0.07)	0.27	
	CON	-0.23	(-0.60, +0.14)	0.27	

Triglycerides (mmol·L^-1^)	HED	0.18	(-0.09, +0.46)^c^	0.22	0.31
	ND	-0.04	(-0.31, +0.23)	0.79	
	VLCHP	-0.11	(-0.32, +0.10)	0.30	
	LCMP	-0.33	(-0.59, -0.06)	<0.05	
	HCLP	-0.13	(-0.30, +0.05)	0.18	
	CON	0.12	(-0.59, +0.33)	0.63	

Insulin (pmol·L^-1^)	HED	1.66	(-0.91, +4.24)	0.23	0.08
	ND	-0.45	(-1.24, +0.33)	0.28	
	VLCHP	-1.23	(-2.15, -0.30)	<0.05	
	LCMP	-0.82	(-2.45, +0.82)	0.33	
	HCLP	-0.10	(-0.61, +0.41)	0.69	
	CON	0.58	(-1.95, +3.12)	0.29	

Glucose (mmol·L^-1^)	HED	-0.12	(-0.42, +0.18)	0.45	0.68
	ND	-0.15	(-0.32, +0.03)	0.12	
	VLCHP	-0.24	(-0.43, -0.04)	<0.05	
	LCMP	-0.08	(-0.42, +0.26)	0.65	
	HCLP	-0.22	(-0.35, -0.10)	<0.001	
	CON	-0.08	(-0.40, +0.25)	0.66	

(HOMA-IR)	HED	0.36	(-0.26, +0.97)	0.28	0.06
	ND	-0.12	(-0.31, +0.07)	0.23	
	VLCHP	-0.35	(-0.58, -0.12)	<0.01	
	LCMP	-0.17	(-0.57, +0.23)	0.40	
	HCLP	-0.06	(-0.19, +0.07)	0.38	
	CON	0.17	(-0.18, +0.52)	0.37	

Leptin (pg·mL^-1^)	HED	-11.4	(-24.7, +2.0)	0.41	<0.01
	ND	-10.5	(-20.2, -0.9)	<0.05	
	VLCHP	-13.9	(-24.0, -3.9)	0.95	
	LCMP	-28.5	(-40.5, -16.5)	<0.001	
	HCLP	-20.6	(-29.3, -12.0)	<0.001	
	CON	24.9	(+1.1, +48.7)	<0.001	

Dietary intake is presented as energy (i.e. calories) intake: % carbohydrate: protein: fat. All data is presented as the mean plus the 95% confidence intervals for the delta response at week 14. Main effects for time are provided as within-group *P*-values. Group × time interaction effects are provided as GxT *P*-values. Significance level was set at 0.05.

^c^Different than LCMP, *P *< 0.05.

### Markers of Fuel Utilization and Energy Regulation

Using serum samples collected at 0 and 14 weeks, serum concentrations of glucose, insulin, leptin, ketones and a homeostatic model assessment for insulin resistance (HOMA-IR) were determined (Table 4) [35]. No significant interactive effects were found for glucose (*P *= 0.68) and ketones (*P *= 0.40), while serum glucose levels were decreased (*P *< 0.001) in the HCLP group. Serum insulin (*P *= 0.08) and HOMA-IR (*P *= 0.06) values approached significance for the HCLP group, but were not significant. Significant within-group reductions in HOMA-IR were found for the VLCHP, but no other groups (Table 4). A significant group × time interaction was found for serum leptin (*P *< 0.05), with significant reductions occurring after the initial phase of dietary restriction. In this respect, leptin levels for all groups except CON (*P *= 0.07) experienced significant reductions (*P *< 0.001 to <0.05) after the first two weeks of dieting, which restricted caloric intake to the greatest extent.

### Clinical Safety Markers

Serum and whole blood safety panels were analyzed from fasting blood after 0 and 14 weeks. While some hematological variables did report main effects over time with no significant interaction effects, none of these changes occurred outside of the clinically accepted normative values for these variables [36] and thus are not being reported. No significant main or interaction effects (*P *> 0.05) were found for found kidney/liver enzymes (e.g. AST, ALT, GGT, Alk Phos) and markers of protein breakdown (e.g. Uric Acid, BUN, creatinine, BUN:creatinine ratio, creatine kinase).

### Psychosocial Results

Individual QOL subscales were completed at baseline and after 10 and 14 weeks and are presented as delta values for 0 and 14 weeks, respectively. Physical functioning (W0: 6.7 ± 22; W14: 9.3 ± 19), bodily pain (W0: 6.1 ± 30; W14: 4.9 ± 25), general health (W0: 8.3 ± 14; W14: 8.4 ± 15), vitality (W0: 11.2 ± 15; W14: 11.9 ± 14), and mental health (W0: 6.3 ± 13; W14: 7.3 ± 14) scores significantly increased (all *P *< 0.05) in exercising individuals (all groups except CON and ND) independent of dietary assignment. Similarly, role emotional scores (W0: -13.2 ± 43; W14: -16.5 ± 44) were significantly (*P *< 0.05) decreased while social functioning scores were unchanged (*P *> 0.05). No significant (*P *> 0.05) correlations were found between the changes in the QOL subscales and anthropometric (e.g. waist circumference) and body composition (e.g. body mass and DXA parameters) variables. Body image assessment revealed significant (*P *< 0.05) percent increases from baseline in exercising individuals irrespective of dietary assignment after 10 and 14 weeks, respectively, for appearance evaluation (W10: 50.5 ± 20; W14: 50.0 ± 20%), appearance orientation (W10: 6.6 ± 32; W14: 6.1 ± 32%), body area satisfaction scale (W10: 23.7 ± 19; W14: 14.7 ± 29%), and overweight preoccupation (W10: 20.1 ± 31; W14: 17.8 ± 34%) while self-classified weight (SCW) did not change. No changes (*P *> 0.05) occurred for any diet or exercise group for the Rosenberg self-esteem scale and the social physique anxiety scale. Changes in DXA fat mass were (*P *< 0.05) correlated with body area satisfaction and self-classified weight scores while changes in percent body fat correlated (*P *< 0.05) with appearance evaluation, appearance orientation, body area satisfaction, self-classified weight and social physique anxiety values.

## Discussion

The purpose of this study was two-fold: 1) to determine the impact of replacing dietary carbohydrate with dietary protein and 2) to determine the impact of the Curves fitness and weight loss program on weight loss, body composition, energy expenditure, psychosocial outcomes, and markers of health in sedentary, obese females. This study represents the first of a series of studies by our research group to examine the effectiveness of the Curves fitness and weight loss program which is currently being followed by millions of women worldwide. Although our study contains many strengths such as our overall sample size, supervised exercise, dietary control measures and inclusion of exercise-only and no exercise/no diet controls, the marked difference in sample size among groups presents some challenges with interpreting our findings. While the authors acknowledge increasing the sample size in these groups would have been helpful, the primary objective was to assess the impact of altering the macronutrient ratio of the dietary regimens while also examining the overall impact of the exercise and diet programs used by the Curves system. Furthermore, statistical power analysis of our primary (e.g. waist circumference) and secondary outcomes (e.g. body mass and DXA body composition variables) ranged from 0.821 – 0.998 with partial eta squared values of 0.062 – 0.115 suggesting that our statistical analysis were adequately powered for our *a priori *determined end points. Our initial hypothesis was that participation in the exercise program would promote weight loss, improve body composition and fitness along with reducing markers of cardiovascular disease and that following a diet which restricted caloric intake while replacing dietary carbohydrate with protein at controlled fat intake levels would result in greater weight loss and improvements in health. Results from this study show that the greatest changes did occur in those groups that restricted their caloric intake in combination with the exercise program while participation in just the exercise program appears to have little to no effect over weight loss and body composition changes, a finding previously reported [8].

Results from the dietary analysis revealed significant differences in energy intake and carbohydrate and protein intake between dietary phases (e.g. Phase I, Phase II, etc.) and dietary groups (e.g. VLCHP and LCMP vs. HCLP). Overall dietary compliance during the 14 week program was deemed successful in all diet groups with the exception of the HED group, where it appears this group struggled to consume their prescribed level of calories (actual mean intake of 1846.6 ± 366.6 kcals·d^1 ^vs. prescribed intake of 2,600 kcals·d^-1^). Caloric intake in this group, however, was higher by an average of 193 kcals·d^-1^, when compared to VLCHP, LCMP and HCLP. This allowed for comparison to lower and caloric intakes while exercising, which was the main premise for this higher dietary prescription, albeit not to the degree we originally hoped. This is likely due to either under-reporting or the inherent error associated with using dietary food records as recent studies from our laboratory have suggested that the magnitude of error associated with self-reporting of food intake is greater when higher caloric intakes are being prescribed [37,38]. An additional important note is that although women in the moderate and high protein diet groups consumed a high percentage of calories in the form of protein, the relative intakes of this macronutrient ranged from 0.9 to 1.1 grams PRO·kg^-1·^d^-1^, respectively, suggesting that overall protein intake was increased above RDA guidelines.

Waist circumference is a key predictor of diabetes and cardiovascular disease [39]. Findings from the present study suggest that participation in a resistance-based circuit exercise program can significantly reduce waist circumference irrespective of what dietary regimen is being followed. Further when dietary carbohydrate is replaced with protein, greater decreases in waist circumference may result (VLCHP:  = -6.3: [-8.7, -3.8]; LCMP:  = -6.7: [-8.7, -4.8]; HCLP:  = -5.7: [-7.5, -3.9]), but these between-group differences were not significant (Figure 2 and Table 2). Regarding weight loss, significant reductions in body mass were found in all groups that followed a diet and participated in the exercise program in amounts similar to recently published investigations [40,41]. As expected, the greatest reductions in body mass occurred in those groups that restricted their calories to the greatest extent and for the longest period of time (VLCHP, LCMP and HCLP), but there were no differences between the amount of lost in the HCLP when compared to VLCHP and LCMP. These findings are consistent with other investigations that investigated the impact of macronutrient content on weight loss in diabetic and non-diabetic populations with no exercise intervention [14,41-43]. Further, findings from Layman [12] suggested that a higher intake of protein with or without exercise was responsible for greater total weight loss [12]. In the present study, participants following the VLCHP and LCMP programs experienced greater but non-significant levels of body mass and fat mass loss when compared to the HCLP group. Similar reductions (all *P *< 0.001) occurred in fat-free mass for VLCHP, LCMP and HCLP, while significant reductions in percent body fat were still found in all three groups. These findings contrast with other studies that have suggested higher protein diets may preferentially help to preserve lean tissue [12,15] in those individuals who have replaced dietary carbohydrate intake with protein. Many reasons exist as to why differences in these findings exist, most notably subtle differences in the diet and exercise interventions. Nevertheless, present findings demonstrate the ability to lose a large percentage of weight as fat while maintaining a large amount of fat free mass while dieting and exercising [7,16] and to a greater extent than other control conditions.

Reductions in energy expenditure have been reported in conjunction with restriction of caloric intake [42,44]. This down-regulation has subsequently been linked to difficulties with weight maintenance as well as regaining the lost body mass over time [8]. Consistent with many prior reports, significant reductions in REE occurred after consuming 1,200 kcal·d^-1 ^for the first two weeks of the present study, even in conjunction with exercise. Caloric intake was increased by 400 kcals·d^1 ^for the next 8 weeks which increased REE back to baseline levels in all groups except HED as this group experienced further increases in REE (Figure 5). A slight increase in energy intake during the following 8 weeks to 1,600 kcals·d^1 ^(which was close to mean daily REE) stimulated increases in REE despite participants losing a significant amount of weight and fat mass. In these groups, REE was further increased when participant's began ingesting a 2,600 kcal: 55:15:30 diet with intermittent dieting (6 days out of 31) at their previously followed energy and macronutrient levels similar to previous energy balance approaches used in the literature [14]. Collectively, these findings suggest that relative levels of energy expenditure can be maintained while completing a weekly resistance-based circuit training program in conjunction with modest caloric restriction (phase II) or potentially even intermittent dieting (Phase III).

As hypothesized, all groups except CON experienced improvements in peak oxygen uptake and maximal strength levels (Table 3) in accordance with other studies which have employed a regular exercise program to overweight/obese and/or sedentary populations [7,40]. Minimal studies have incorporated a circuit-style resistance mode of exercise to this population, thus providing evidence that this type of training promotes general improvements in fitness in this population. Additionally, the serum reductions in glucose (2.6%), total cholesterol (2.5%) and LDL cholesterol (2.8%) were significant and similar to other studies which have employed dietary regimens that replace dietary carbohydrate in favor of greater dietary protein (Table 4) [45]. While no significant group × time effect was found for insulin (*P *= 0.08), individuals in the VLCHP and LCMP groups experienced significant reductions, providing continued support that higher intakes of protein may help to promote such changes [14]. A weakness of our study design is the inherent nature of pneumatic resistance. Such exercise modes make it impossible to determine how much resistance is actually being applied and subsequently creates challenges for documenting changes in total work throughout the exercise program in all groups. In this regard, it is possible that the total work completed from workout to workout and week to week didn't change or even decreased, although the physiological adaptations which did occur make this unlikely. For this reason, heart rate and ratings of perceived exertion were monitored throughout every exercise session in an attempt to promote consistency from workout to workout, while the number of completed repetitions were recorded to promote adequate progression. It was suggested that as fitness parameters increased each individual's ability to complete work would also be subsequently increased. Although each workout was intermittent in nature, heart rate monitoring did successfully keep individual workouts in the same relative range of exercise intensity

The relationship of leptin to weight loss, energy expenditure and insulin sensitivity has been characterized [3,46,47]. In this regard, circulating levels of leptin have been shown to decrease in response to decreases in energy availability [46], however, the influence of exercise and alterations in the macronutrient ratio is still undetermined. Volek and colleagues [3] suggested that significant decreases in leptin occur as part of an 8-week weight loss program, which similarly occurred in the present study. Further, Sartorio et al. [46] used a combination of energy-restricted diets with a 5 d·wk^-1 ^aerobic and anaerobic exercise program and reported an acute, significant reduction in leptin levels which closely mimicked changes in body mass. As observed in the Sartorio study, serum leptin changes in the present study mimicked body mass changes and significantly decreased in all groups (except CON) after 2 weeks (Table 4). No significant changes occurred in any diet or exercise groups for markers of kidney and liver function (e.g. AST, ALT, GGT, creatinine, etc.) and fat or protein breakdown (e.g. ketones, total protein, BUN, etc.). Present findings support contentions that higher proteins do not invoke negative alterations in any of these serum variables after 14 weeks in obese, but otherwise health populations [1,3,43,45].

An additional area of importance relative to public health and exercise adherence were the psychosocial assessments. In this regard, individual subscales of the SF-36 (e.g. physical functioning, bodily pain, general health, vitality, and mental health) significantly improved throughout exercise. While it could be stated that the significant increase in the bodily pain subscale was a negative response, it is an expected response considering the participants were sedentary for an extended period of time prior to the start of the study and placed in a resistance-training program, likely invoking some stiffness and delayed-onset muscle soreness. Additionally, significant increases in several subscales (e.g. appearance evaluation, appearance orientation, body area satisfaction and overweight preoccupation) of body image evaluation also improved in those individuals who were following the exercise program. In addition to improvement in several of the psychosocial variables, changes in these variables were significantly (*P *< 0.05) correlated to changes in body composition (e.g. fat mass and body fat %). Considering statistical probability that one of every twenty correlations run will result in a significant finding, it is possible that significance was found merely due to chance. While not intended to be causal, these findings suggest that changes in body composition may operate in conjunction with changes in body image as a result of exercise participation.

As such our investigation has many strengths and weaknesses to consider when evaluating this data against other data in this research area. Our study design could have been more complete by including a diet only control group with restricted caloric intake as this would have allowed us to more closely evaluate the impact of the exercise program in each dietary group. In this respect, a recent study has illustrated a similar weight-loss effect when a dieting only approach or a combination of exercise and diet is used if the net shift in energy expenditure is the same, however, a greater fitness benefit was reported which could have implications for cardiovascular health [40]. Additionally, while the sample size across groups varied widely, our primary interest was in evaluating the changes in different dietary regimens with the exercise program. Strengths in our study involve the use of a rather large sample over several weeks of the intervention in comparison to other related studies. Also, a strength in the present study is our examination of a commercial program that is available to women all over the world and is currently the program of choice for millions of women at over 10,000 locations across the globe.

## Conclusion

In summary, results of this study indicate that combining a diet that restricts caloric intake in combination with a resistance-based circuit exercise program stimulates the greatest amount of weight loss and improvements in measures of body composition (e.g. waist circumference, DXA, etc.). When carbohydrate is replaced with protein while keeping fat intake at recommended levels (VLCHP and LCMP), larger decreases in waist circumference, body mass, fat mass and fat-free mass when compared to a diet that has a higher proportion of carbohydrate (HCLP) in addition to greater decreases in fasting insulin levels. As expected, regular participation in a resistance-based circuit exercise program resulted in marked losses of body mass, improvements in body composition and overall improvements in cardiovascular and musculoskeletal fitness. Participation in the exercise program allowed for weight loss without concomitant reductions in resting energy expenditure. Additionally, those participants who were identified to have a low resting energy expenditure at baseline also experienced weight loss while exercising, but being prescribed a diet higher in calories. Significant reductions in leptin levels as well as improvements in fitness, markers of health, health-related quality of life, and body image were found for all individuals who followed the exercise program. These findings suggest that replacing carbohydrate with protein can be an effective strategy to improve body composition and reduce cardiovascular disease markers while participating in a resistance-based circuit exercise program in sedentary overweight women.

## Abbreviations

HED: High-energy diet + exercise group; VLCHP: Very low carbohydrate, high protein diet + exercise group; LCMP: Low calorie, moderate protein diet + exercise group; HCLP: High carbohydrate, low protein diet + exercise group; CON: Control group; ND: No diet + exercise group; U·L^-1^: units per liter; mmol·L^-1^: millimoles per liter; μmol·L^-1^: micromoles per liter; pmol·L^-1^: picomoles per liter; mM: millimoles; DXA: dual energy x-ray absorptiometry; HOMA-IR: homeostatic model assessment of insulin resistance.

## Competing interests

Curves International (Waco, Texas, USA) provided funding for this project through an unrestricted grant to Richard Kreider, PhD while at Baylor University. All researchers involved independently collected, analyzed, and interpreted the results from this study and have no financial interests concerning the outcome of this investigation. Publication of these findings should not be viewed as endorsement by the investigators, their institutions, or the editorial board of Nutrition and Metabolism.

## Authors' contributions

CK: Coordinated study, assisted with data collection and analysis, completed statistical analysis and prepared manuscript.

AT: Assisted with data collection and analysis. Coordinated all dietary aspects of study.

BC, LT, CW, BM, MR, EP, MG, JO: Assisted with recruitment, compliance, data collection and analysis.

TMC: Assisted with data collection and analysis. Coordinated study.

CR: Coordinator of all laboratory staff and studies. Assisted with data collection and analysis.

RW: Served as medical director and provided medical clearance for all participants.

RK: Designed study and procured grant funding. Oversaw all aspects of study and assisted with statistical analysis and preparing manuscript.
